# Dual Primary Malignancy: A Rare Organ Combination

**DOI:** 10.1155/2014/760631

**Published:** 2014-10-07

**Authors:** Preetam Acharya, Anand Ramakrishna, Tanuj Kanchan, Rahul Magazine

**Affiliations:** ^1^Department of Pulmonary Medicine, Kasturba Medical College-Mangalore, Manipal University, Manipal, Karnataka 575 001, India; ^2^Department of Forensic Medicine and Toxicology, Kasturba Medical College-Mangalore, Manipal University, Manipal, Karnataka 575 001, India; ^3^Department of Pulmonary Medicine, Kasturba Medical College-Manipal, Manipal University, Manipal, Karnataka 576 104, India

## Abstract

A 63-year-old female smoker was evaluated for lump over the right breast, fine needle aspiration cytology of which showed infiltrating ductal carcinoma. Investigations also revealed the presence of left upper lobe mass lesion, the biopsy of which suggested small cell carcinoma. The existence of two malignancies having different histopathologies at anatomically distinct sites suggests the diagnosis of dual primary malignancy involving the breast and the lung which, being a rare combination, prompted us to report the case.

## 1. Introduction

The presence of multiple primary cancers in a single patient was first reported more than 100 years back. Since then, this phenomenon has been identified with increasing frequency, in part due to the increase in life expectancy of cancer survivors—a boon of advancements in cancer therapeutics—and to the more comprehensive screening protocols used in cancer patients. The two cancers are either detected at the same time (synchronous) or one may follow the other after a period of time (metachronous). Although breast and lung cancers are one of the commonest tumors in females, yet a synchronously existing primary involving both organs in a single patient is a rarity in medical literature, prompting us to publish this case.

## 2. Case Report

**Figure 1:**
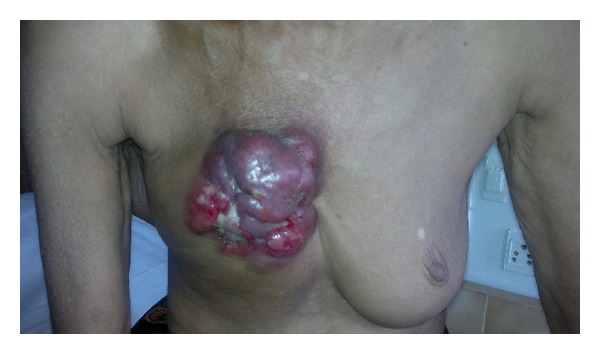
Photomicrograph of polypoid mass over the right breast.

**Figure 2:**
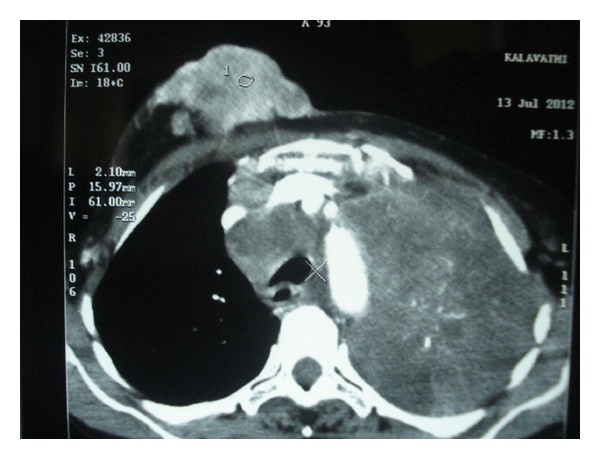
Contrast-enhanced computed tomography (CECT) chest showing left upper lobe lung mass with collapse and left mild pleural effusion. Irregular mass seen in the right breast with loss of fat planes between it and the chest wall.

**Figure 3:**
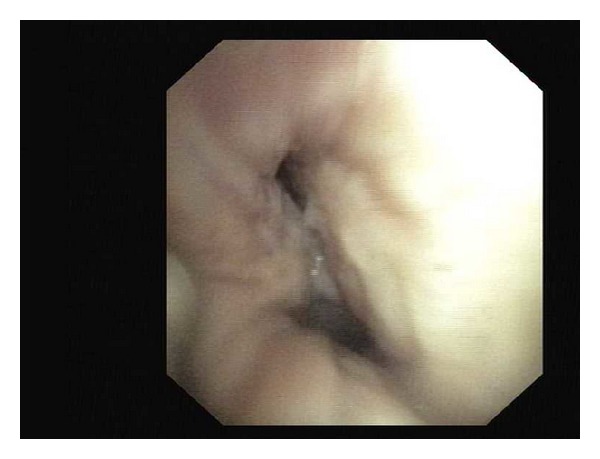
Bronchoscopy image showing concentric narrowing of left mainstem bronchus with mucosal irregularity.

A 63-year-old female patient was admitted for evaluation of a lump over the right breast since 15 months which was painless and had gradually increased in size over the last 3 months. She also had anorexia with significant weight loss. She complained of progressively worsening shortness of breath (from Modified Medical Research Council (MMRC) Grade I to Grade III) over last 2 months. There was no other respiratory complaint. She denied any history of fever or night sweats. Patient was occupied in the beedi-rolling industry and used to smoke beedis (a hand-rolled Indian cigarette). There was no history of alcohol intake. She did not have any family history of breast or ovarian cancer. She had been postmenopausal since the last 8 years and did not give history of oral contraceptive pill (OCP) use. On examination, a polypoid mass measuring approximately 6 cm × 4 cm was seen over the right upper quadrant of the breast (Figure 1). The mass was hard in consistency, nontender, and freely mobile over the chest wall with fixity to the skin above. The skin overlying the lump was ulcerated at places with serosanguineous discharge. There was no nipple or areolar discharge. Supraclavicular lymph nodes were palpable bilaterally along with palpable anterior axillary lymph nodes. Respiratory system auscultation revealed reduced breath sounds over the left hemithorax with a monophonic wheeze. Basic blood investigations were normal except for an elevated ESR (105 mm/1st hour). A chest radiograph showed a left upper lobe collapse with mild left pleural effusion. A contrast-enhanced computed tomography of thorax (CECT) revealed a soft tissue density mass lesion involving upper lobe of left lung, compressing the trachea and causing cutoff of the left upper lobe bronchus, leading to its collapse. In addition, an irregularly enhancing soft tissue density mass lesion measuring 8 cm × 7.9 cm × 4 cm was noted in the right breast with loss of fat planes between lesion and right pectoralis muscle (Figure 2). Fine needle aspiration was done from the breast lump which was suggestive of infiltrating ductal carcinoma. Bronchoscopic examination showed narrowing of left mainstem bronchus with mucosal infiltration (Figure 3). Histopathological examination of bronchial biopsy specimen showed features suggestive of small cell carcinoma of the lung. A diagnosis of a synchronous dual primary cancer of lung and breast was made. The patient was advised referral to the Cancer Institute for further investigations and initiating chemotherapy for her lung tumor and hormonal manipulation based on ER, PR, HER-2 markers for her breast cancer which she refused. She was given the best supportive care keeping in mind the advanced stage of both malignancies and her general physical condition and expired at home shortly after discharge.

## 3. Discussion

Multiple primary malignancies in a single patient were first described in 1879 by Billroth [1]. The neoplasms may be limited to a single organ or, as in our case, involve multiple and anatomically separate organs. The North American Association of Central Cancer Registries (NAACCR) classifies multiple primary tumors into two categories: (1)* Synchronous*, in which the cancers occur at the same time (the Surveillance Epidemiology and End Results Program (SEER) definition is within two months) and (2)* Metachronous*, in which the cancers follow in sequence, that is, more than two months apart [2]. Metachronous primary malignancies are becoming increasingly common because of an increase in the number of elderly cancer survivors, greater awareness, and improved diagnostic modalities. In comparison, synchronous tumors occur uncommonly, with the most common site for synchronously existing multiple tumors being the breast.

Whether the second lesion is truly a primary or represents metastases is difficult to decide and for this the Warren and Gates criteria (1932) are used which proposed that a diagnosis of multiple primary malignancies requires the following [1]:

1. each tumor should present a definite picture of malignancy;
2. each tumor should be histologically distinct;
3. the possibility that one is a metastasis of the other must be excluded.

A study analyzing the SEER Program database has revealed that the incidence of multiple primaries varies from 1% (initial liver primary) to as high as 16% (initial bladder primary); if initial primary is the breast, the percentage of patients expected to develop multiple primaries is 10% and for the lung it is 4% [3]. Meta-analyses show the frequency of second primary tumor (SPT) as 3–5%, third tumor (TT) as 0.5%, and fourth tumor, that is, quadrant tumor (QT), as 0.3%, in different organ and of different histogenesis [4]. Liu et al. reported that the most common tumors accompanying lung cancer were in the aerodigestive tract (in descending order of frequency: larynx, nasopharynx, esophagus, oral cavity, and hypopharynx), followed by colorectal and cervical malignancies [5].

The pathophysiology behind the occurrence of multiple primary malignancies has been theorized to be common-carcinogen induced multiple cancers in an exposed epithelial surface, called “field-cancerization” as seen in head-neck tumors, as a late side effect of treatment used to treat the first tumor, and a genetic predisposition to neoplasia [6]. Other possible causal factors include persistent carcinogen exposure from environment, progressive ozone depletion and effects of ionizing radiation, increased use of organ transplant, and the increasing use of newer treatment modalities like hormonal manipulation, target therapies, genetic manipulation, and immunomodulators [7].

An autopsy series has reported that a greater percentage of multiple primary cancers occur in the same organ or in organs of the same system rather than in unrelated organs [8]. Breast and lung cancers are the two most frequent cancers detected in women, yet a double primary cancer involving the two has been reported only rarely in the literature. Kurishima et al. in their study of 98 lung cancer patients having synchronous and/or metachronous malignancies did not report even a single patient with breast cancer as the second tumor [9].

Differential diagnosis for the patient in our case included lung metastases from the breast primary or vice versa. Breast metastases from extra mammary primary neoplasms are very rare, accounting for about 2% of all breast malignancies [10]. The lung, though, is a common site of metastasis for breast cancer, with one study showing nearly a 30% incidence of patients with breast cancer developing a lung metastasis [11]. Double primary cancer is a more reasonable diagnosis in this case since the histopathology of the breast lump was an infiltrating ductal carcinoma and that of the lung mass was small cell carcinoma thus ruling out any possibility of metastasis from one site to the other. This assumption is also in agreement with the NAACCR definition that “*multiple lesions of different histologic types occurring in different sites are considered as separate primaries whether occurring simultaneously or at different times*” [2].

The occurrence of dual primary malignancies is not rare. With lung cancer, finding a tobacco related cancer like cancer of larynx and bladder is possible. In this patient, the presence of small cell lung cancer may be explained by smoking and exposure to domestic biofuels, but these do not account for breast cancer, a nontobacco associated neoplasm. This rare coexistence of lung with breast cancer may therefore be due to coincidence, which prompted us to report this case.

In conclusion, this case highlights the fact that the presence of a lesion anatomically away from the primary malignancy should be labelled as a metastasis only after detailed evaluation; otherwise, there is a possibility of missing a synchronous primary malignancy.
